# A Potential Animal Model of Maladaptive Palatable Food Consumption Followed by Delayed Discomfort

**DOI:** 10.3389/fnins.2017.00377

**Published:** 2017-07-05

**Authors:** Lital Moshe, Liza Bekker, Aron Weller

**Affiliations:** ^1^Department of Psychology, Bar-Ilan UniversityRamat-Gan, Israel; ^2^Developmental Psychobiology Lab, Gonda Brain Research Center, Bar-Ilan UniversityRamat-Gan, Israel

**Keywords:** binge-eating, delayed aversive consequences, hedonic eating, abdominal discomfort, lactose, rats, reward, animal model

## Abstract

**Introduction:** Binging is the consumption of larger amounts of food in a briefer period of time than would normally be consumed under similar circumstances. Binging requires palatable food (PF) to trigger abnormal eating, probably reflecting gene × environment interactions. In this study we examined the impact of trait binge eating (BE) and its compulsive nature on the conflict between hedonic eating of PF and anticipation of a delayed aversive effect. We used female rats as an animal model similar to other models of BE. A novel aspect of this model in this paper is the use of a delayed internal aversive effect produced by lactose ingestion. Establishing this model will allow us to better understand the nature of the conflict between immediate reward and its delayed aversive implications. We hypothesized that BE prone (BEP) rats will demonstrate maladaptive decision making, presenting higher motivation toward PF even when this is associated with delayed discomfort.

**Method:** (Phase 1) 52 female adult Wistar rats were divided to two eating profiles: resistant and prone binge eaters (BER/BEP) based on intake of liquid PF (Ensure). Next, all subjects underwent a Lactose Conditioning Protocol (LCP) that included 4 h tests, one baseline and 3 conditioning days (Phase 2), in which solid PF (Oreo cookies) was paired with glucose (control-no internal aversive effect) or lactose, dissolved in liquid PF. Index for PF motivation was PF consumption during the 4 h LCP. To test for memory of lactose conditioning, we performed another LCP with glucose only (anticipation, but no actual lactose-induced discomfort), a week after the last conditioning session.

**Results:** Lactose conditioned BEP showed higher motivation toward PF compared to lactose conditioned BER faced with delayed aversive effects. Only lactose conditioned BER rats devaluated the PF over LCP days, indicating an association between PF and abdominal discomfort. In addition, only lactose conditioned BER presented an adaptive dynamic behavior, by varying PF intake according to consequences. Furthermore, solid PF consumption was predicted by binge size of liquid PF, only for lactose conditioned rats.

**Conclusions**: We established an animal model for a common eating conflict in humans using delayed internal aversive unconditional stimuli.

## Introduction

Many people are struggling with the conflict between the pleasure of sweet and/or high caloric food and its later, and sometime cumulative, consequences. What makes some of us better at making the adaptive choice? This study will focus on palatable food (PF) intake in a conflict situation, while assessing the impact of “trait binge eating.”

Binge-eating (BE or “compulsive eating”) is described as the consumption of larger amounts of food, in a brief period of time than would normally be consumed under similar circumstances (APA, 2013). Binging usually includes highly palatable foods that are typically characterized by high calorie density and low nutritional values (Oswald et al., 2011; Bekker et al., 2014; Boggiano et al., 2014; Pool et al., 2015) and it is often accompanied by a sense of loss of control in humans (Finlayson et al., 2011; APA, 2013; Boggiano et al., 2014, 2015; Dalton and Finlayson, 2014; Pool et al., 2015) which is triggered by emotional stressors (Boggiano et al., 2007, 2014, 2015; Bello et al., 2014; Pool et al., 2015). Food consumption while binging is not necessarily driven by hunger and therefore it increases various health risks (Finlayson et al., 2011; APA, 2013; Bekker et al., 2014; Boggiano et al., 2015; Imperatori et al., 2015; Pool et al., 2015). Behavioral, emotional, and cognitive properties of BE are seen in 10–20% of the general population (Dalton and Finlayson, 2014). In rats, trait BE-like behavior has been characterized by BE prone (BEP) vs. BE resistant (BER) behavioral profiles (Boggiano et al., 2007).

It was previously shown that persistent overeating can lead to a pattern of compulsion behavior such as binging. Compulsive eating behavior indicates pathological motivation toward food (Ventura et al., 2013). Adaptive decision making refers to the process of choosing a particular behavior over alternatives under the presumption that this behavior will produce the most beneficial outcome (Kim and Lee, 2011). For some, food may become a superior reward, so that its consumption continues despite awareness of negative health consequences. The severity of BED depends on behaviors such as eating until becoming nauseous or eating in secret, and cognitions such as the sense of loss of control or guilt feelings after a meal. In this case, there is a lack of behavioral flexibility that prevents adaptive changing in coping (Bickel et al., 2012). Similar to addiction disorders (Curtis and Davis, 2014), compulsive behaviors represent a loss of control over habits, which creates pathological courses of behaviors (Jentsch et al., 2014). Impairment in the reward system can change decision making and learning through the Reward Prediction Error (RPE) that includes the pros and cons of the behavioral outcomes (Benton, 2010; Hauser et al., 2017). The study of Oswald et al. (2011) investigated, in rats, the maladaptive situation in which repeated intake of PF occurs along with knowledge that an aversive outcome is likely to follow. With the use of incrementing levels of electrical foot shock delivered immediately after retrieval of the PF, these researchers showed the compulsive nature of eating PF despite aversive consequences, in satiated BEP rats.

The nutritional state of the organism influences the regulation of feeding and decisions related to food (Hilbert and Kim, 2017). Food intake, eating behaviors, and decision making are regulated by peptide hormones such as ghrelin, leptin, and insulin (Murray et al., 2014; Anderberg et al., 2015; Hilbert and Kim, 2017), at least partially through their influence on dopamine metabolites (Murray et al., 2014). Dopamine (DA) is a main neurotransmitter involved in reward and eating motivation (Bello and Hajnal, 2010; Corwin et al., 2011) and it is implicated in motivated reward seeking (Benton, 2010) and avoidance behaviors (Umberg and Pothos, 2011; Baarendse and Vanderschuren, 2012). In binge eating rats, type 2 DA receptor antagonist in PFC increased palatable food intake while a D2R agonist decreased PF consumption (Corwin et al., 2016). Furthermore, in VTA, dopamine-related gene expression of tyrosine hydroxylase, the dopamine transporter, and the D2 receptor was higher in BE rats assessed before the binge episode (Corwin et al., 2016). Overeating can be explained, at least in part, in relation to the interaction between hormones and the reward system (Murray et al., 2014). For example, leptin acts in the ventral tegmental area (VTA) and the lateral hypothalamus (LHA) to decrease food intake through its influence on dopamine metabolites. Insulin also acts in the VTA via dopaminergic neurons by up-regulating dopamine transporter (DAT) activity. Ghrelin receptor expression was also found in the reward system, on GABAergic and dopaminergic neurons in the VTA and the substantia nigra (SN), and ghrelin activates dopamine and acetylcholine receptors in the VTA and nucleus accumbens (NAc; Murray et al., 2014). Intake of palatable foods elicits dopamine release from cells originating in the VTA, activating the NAc via the medial forebrain bundles (Roh et al., 2016). In contrast, acetylcholine (Ach) correlates with slow onset and maintenance of aversion, reflecting changes in reward evaluation. Ach and DA have contrasting influences on the reward of food in the NAc. Endogenous appetite suppressants release Ach in the NAc, meaning that the rewarding aspect of eating may potentially become aversive when satiation arrives. Therefore, PF intake will cause release of DA or Ach depending on learned expectations (Umberg and Pothos, 2011). Dopamine levels decrease as the threshold for reward stimuli rises (Umberg and Pothos, 2011), demonstrating the Reward Deficiency Syndrome (RDS) that is characterized by hyper function of dopamine and abnormal craving behavior (Blum et al., 2014), as seen in binge eating.

Binge eaters have a relatively high expression of the mu-opioid receptor gene, which correlates with higher scores on a self-report measure of hedonic eating (Fantino et al., 1986; Kelley et al., 2003; Davis et al., 2009; Corwin et al., 2011). Opioid peptides within the ventral striatum regulate the affective response to highly palatable and energy-dense foods by increasing the perceived palatability of food (Grigson, 2002; Kelley et al., 2003; Benton, 2010). High-fat diet appears to prime the brain to binging by sensitization of opioid-receptors (Hagan and Moss, 1991) which influences the DA/Ach balance (Umberg and Pothos, 2011). Binge-eating prone rats consumed as much palatable food when sated as when hungry, which may reflect an altered or higher hedonic threshold or heightened reward sensitivity (Boggiano et al., 2007).

The main goal of the current study is to form a valid animal model that will enable to simulate PF intake conflict in the presence of unwanted implications. In this first implementation of the model, we strive to identify characteristics that differ between prone binge eaters, and resistant binge eaters in aspects that affect PF eating behavior in the presence of aversive circumstances. The model presented in this study is based on Delayed Reward Discounting (DRD) that represents the tendency to prefer small immediate reward over a delayed but more beneficial reward (Baarendse and Vanderschuren, 2012; Jupp and Dalley, 2014; Anderberg et al., 2015; Schippers et al., 2016). The main difference from the classic task of DRD is the change in value of the immediate reward by its prediction of a later negative event. In this study we will use the approach lead by Corwin et al. (2011) in which the PF intake is considered an index of BE. The innovation of the current study is the use of a delayed negative impact and the use of internal discomfort as the negative consequence. As opposed to other studies that used external negative effect such as electrical shock or interceptive pain induced by LiC1 (lithium chloride that causes instant nausea), we chose these two critical qualities in order to simulate more real life scenarios in which the consequences of our actions, practically toward food, are not always immediate or external. Similarly, when on a diet, one who wants to lose weight will see results only days, weeks, or months after avoiding high fat food.

The type of internal aversive stimulus used in the current study is lower abdominal discomfort caused by acute lactose ingestion. The use of lactose is based on insufficient lactase activity (the enzyme that breaks down lactose) in the adult rat's digestive system. When lactose enters the digestive system and there is not enough lactase activity available, it draws water through osmosis and as a result causes diarrhea, cramping, intestinal gas, and intestinal hyperactivity (Mir and Alioto, 1982; Simbayi et al., 1986; Smith and James, 1987; Liuzzi et al., 1998; van de Heijning et al., 2015). There is an important difference between food rejection based on taste aversion and food rejection based on an expectation for a negative digestive response. Results from a number of studies show that distress caused in the upper abdomen, such as nausea caused by LiC1, affects taste evaluation, unlike distress caused in the lower abdomen or negative impact from external stimuli that decreases eating due to expectation for a negative effect (Pelchat et al., 1983; Simbayi et al., 1986; DiBattista, 1990, 1992; Lin et al., 2014). This study to our best knowledge is the first to examine the effect of a delayed internal aversive stimulus on PF consumption in the context of eating disorders.

We hypothesize that prone binge eaters will demonstrate maladaptive decision making and will present high motivation toward PF even when anticipating a familiar internal aversive effect, associated with PF consumption. We also hypothesize that this behavioral model can be used as a basis for studying the neurobiological mechanisms underlying impulsive choices and individual differences in conflict-related decision making.

## Methods

This study was carried out in accordance with the recommendations of the Society for Neuroscience. The protocol was approved by Bar Ilan University's Institutional Animal Care and Use Committee. Protocol number: 21-03-2016.

### Animals

Fifty two female adult Wistar rats (*M* = 106 days old, SE = 7.5, Mean body weight 230.67 ± 19.5 g.) were housed in pairs in standard cages (18.5 cm height × 26.5 cm width × 43 cm length) containing a plastic tube, in a room with an adjusted light-dark cycle (lights off 13:00–01:00 h) and controlled temperature (20°–24°). Experiments started 1 h before dark and continued during the dark portion of the cycle. Before the beginning of the experiment, the rats underwent a Safe taste acquisition protocol (Jan and Bowman, 1974; Siegel et al., 1974; Best, 1975; Lin et al., 2014) to the PFs of the study (Ensure, Oreo cookies and glucose). The protocol allowed free access to the PFs in the home cage 3 days before the onset of the test.

### Pilot study: lactose moderate effect

To assess if 5 g of lactose per 100 g body weight (Pelchat et al., 1983) indeed produces moderate discomfort that will affect behavior without causing prolonged pain or sickness, a pilot study with a small sample of rats (*n* = 8) was performed in which they were carefully observed after ingesting 3 ml of Vanilla Ensure Plus (Abbott Nutrition, 1.5 kcal per gram) containing glucose for 2 test days and lactose for 2 test days with interval of 1–2 days between test days. Each test day included 4 h of observation after sugar ingestion in which the experimenter observed the rat's behavior for 5 min every 20–30 min. Observations looking for aversive behaviors were made throughout all experiments (as in Pelchat et al., 1983; Simbayi et al., 1986) Specific aversive expressions that would have required special attention were: Gasp, chin rubbing, head shaking, and paw waving. Results: No behavioral signs of discomfort or pain were observed; loose stools were also not observed. Data concerning body weight and food intake were noted throughout all experiments and no negative effects of lactose were noted.

### Experimental time line

**Figure 1:**
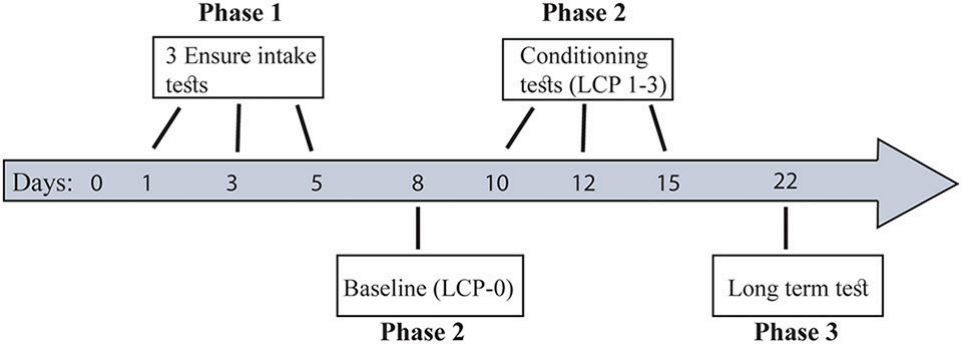
Study timeline. Phase 1: Eating pattern division to BER and BEP profiles. Phase 2: Lactose ingestion protocol and PF consumption. Phase 3: Long term test-conditioned-lactose memory. During all tests days, the rats had free access to water and chow at all times.

Briefly (for more details see Figure 1), all rats underwent 3 Ensure intake tests (Phase 1) in order to divide them into two eating profiles: binge eating resistant (BER) and binge eating prone (BEP). Next, the rats from the two eating profiles were subjected to a lactose conditioning protocol (LCP) during which they were divided to either the lactose or the glucose group (Phase 2). At this phase we used Oreo cookies (made by Nabisco, 4.7 kcal per gram) consumption to assess binge size. The first LCP day (LCP-0) served as baseline, to assess Oreo cookie intake after exposure to glucose (without lactose administration). Next the rats underwent 3 LCP tests of Oreo cookie consumption after exposure to lactose/glucose with 1–2 days interval between tests. To test the existence of long term memory for conditioned lactose impact, another LCP test was performed a week later (Phase 3) without lactose administration (only glucose preceded the Oreo cookie intake session). During all tests days, the rats had free access to water and chow at all times. Animals were separated to single-rat cages for the time of the tests solely and returned to their pair home cages after each test.

### Phase 1: eating pattern division for BER and BEP

Rats were given 2 h access (between 12:00 and 14:00 h) to Vanilla Ensure Plus (Abbott Nutrition) for 3 days, 48 h apart in individual cages. As in our previous studies (Bekker et al., 2014; Barnea et al., 2017), the rats were not deprived before these tests. Based on the rank-order of the intake of Ensure eaten during the 2 h tests (normalized to body weight), the rats were divided as in Boggiano et al. (2007) to BER and BEP eating profile groups by the lower and higher tertiles, respectively.

### Phase 2: lactose ingestion and PF consumption

This phase includes 1 baseline and 3 conditioning sessions pairing PF consumption with glucose or lactose. The conditioning was limited to three sessions to prevent the acquisition of a conditioned taste aversion over time. The test days were 48–72 h apart.

### Baseline (LCP-0)

On the first LCP day, all rats were first placed in an individual empty test cage with a plate containing solution of 3 ml Ensure and glucose (5/100 g body weight; Pelchat et al., 1983). When the rat finished the solution or 60 min had elapsed, the rat was removed from the empty test cage and placed in a regular test cage with bedding, tube, water, chow and Oreo cookies for 4 h. Measures of chow and Oreo cookie intake were noted after 2 and 4 h (Boggiano et al., 2007).

### LCP tests

Rats from both conditioning groups (lactose/glucose) were first placed in an individual empty test cage as in baseline, however, half were given a plate containing solution of 3 ml Ensure and lactose and half were provided with 3 ml Ensure and glucose as the control sugar (5/100 g body weight; Pelchat et al., 1983), respectively within BER and BEP groups. Terms for rats' removal from the empty cage to the regular cage and measures of PF and chow remained as in baseline.

### Phase 3: long term test-conditioned-lactose memory

This test day was performed a week from the last LCP day in order to identify long term memory for the PF condition. On this day, rats went through the same procedure as in the baseline day with no lactose administrated.

### Statistical analyses

All intake measures (Ensure, Oreo cookies, and Chow) were normalized to body weight using the Heusner formula [kCal/(Body Weight^∧^0.66) Heusner, 1985]. PF preference was calculated as the percent Oreo cookie intake in calories (normalized to BW) from the total caloric intake (cookies + chow). Consumptions of PF and chow were calculated at two time points: first and last 2 h. Last 2 h consumption was calculated by reduction of first 2 h consumption from total intake.

The effects of the eating profile and the conditioning sessions on the rats' preference and consumption were analyzed using the relevant statistic test (*t*-test, two way analysis of variance (ANOVA), ANOVA with repeated measures or linear regression).

## Results

### Phase 1: eating profile division for BER and BEP

**Figure 2:**
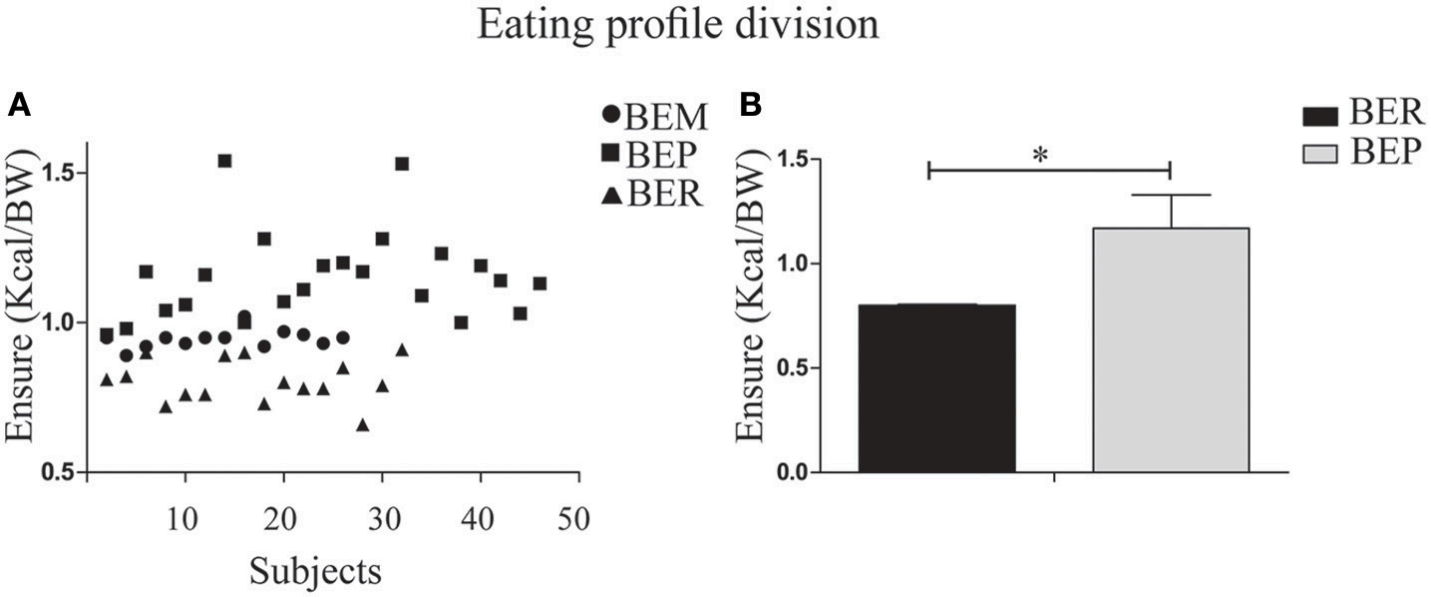
Division of the rat population into BER/BEP status groups. **(A)** Subject scatter by mean Ensure PF intake over the second and third days of Phase 1. The x-axis represents the entire sample. BEM (binge eating middle third) rats were excluded from analysis. The lower tertile was determined as BER (mean PFcn ± SEM = 0.8 ± 0.07) and the upper tertile was determined as BEP (1.17 ± 0.14). **(B)** Mean Ensure PF intake for BER and BEP eating profile groups. ^*^p < 0.05, ^**^p < 0.005, ^***^p < 0.001.

Segmentation of the rats into status groups by thirds, based on the Vanilla Ensure Plus intake, normalized to BW (using the Heusner formula; Heusner, 1985) was employed to characterize the individual animals as BER or BEP. This was performed for the second and third day of Phase 1 in all subjects. The lower third was determined as BER (mean = 0.8 ± 0.07, *n* = 16) and the upper third was determined as BEP (1.17 ± 0.14, *n* = 23). The middle third (BEM) were excluded from the analysis (Figure 2).

### Phase 2: lactose conditioning protocol (LCP) and PF consumption

#### Baseline day (LCP-0)

**Figure 3:**
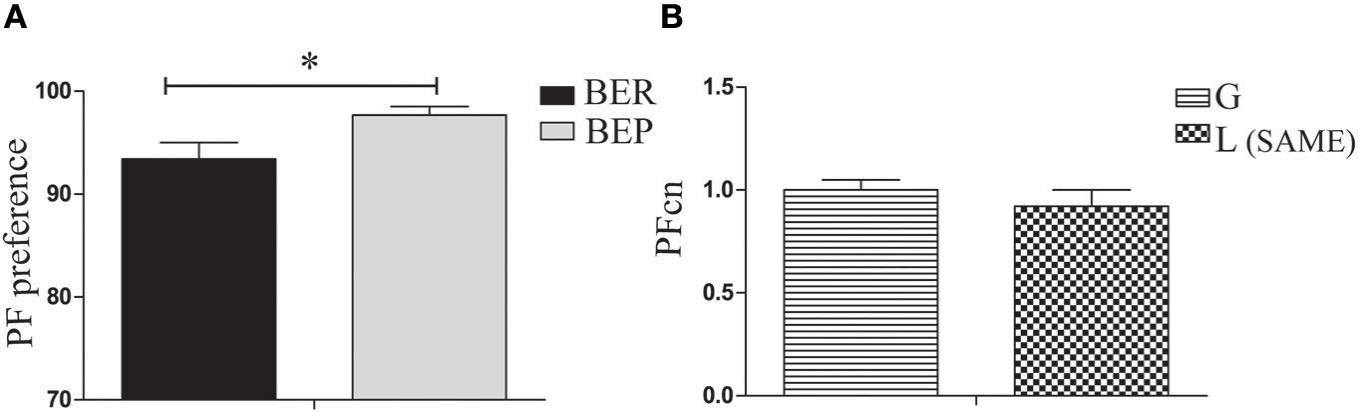
PF consumption during LCP-0. **(A)** Gap in PF intake between BER and BEP was maintained throughout phases. PF preference was calculated as the percent of PF caloric intake relative to total caloric intake during the 4 h test. BEP rats consumed significantly more PF than BER rats. **(B)** There were no significant differences in PFcn between rats that will be conditioned with glucose or with lactose. ^*^p < 0.05, ^**^p < 0.005, ^***^p < 0.001.

The aim of the LCP-0 day was to expose the rats to the sequence of conditioning tests in which Oreo cookies are available side by side with chow in a regular cage for 4 h, after finishing their solution in the empty cage. On this day, all rats received glucose in their Ensure solution. Results show that eating profiles remained stable as was determined in Phase 1 (BER and BEP), with a significant difference between them in PF preference [*t*_(38)_ = 2.55, *p* = 0.031, Figure 3A]. In addition, there were no significant differences in the PFcn (palatable food consumption normalized to body weight) between rats to be conditioned during LCP-1-3 with glucose and those to be conditioned with lactose [*t*_(49)_ = 0.81, *p* = n.s, Figure 3B].

### Lactose conditioning protocol (LCP: 1–3)

**Figure 4:**
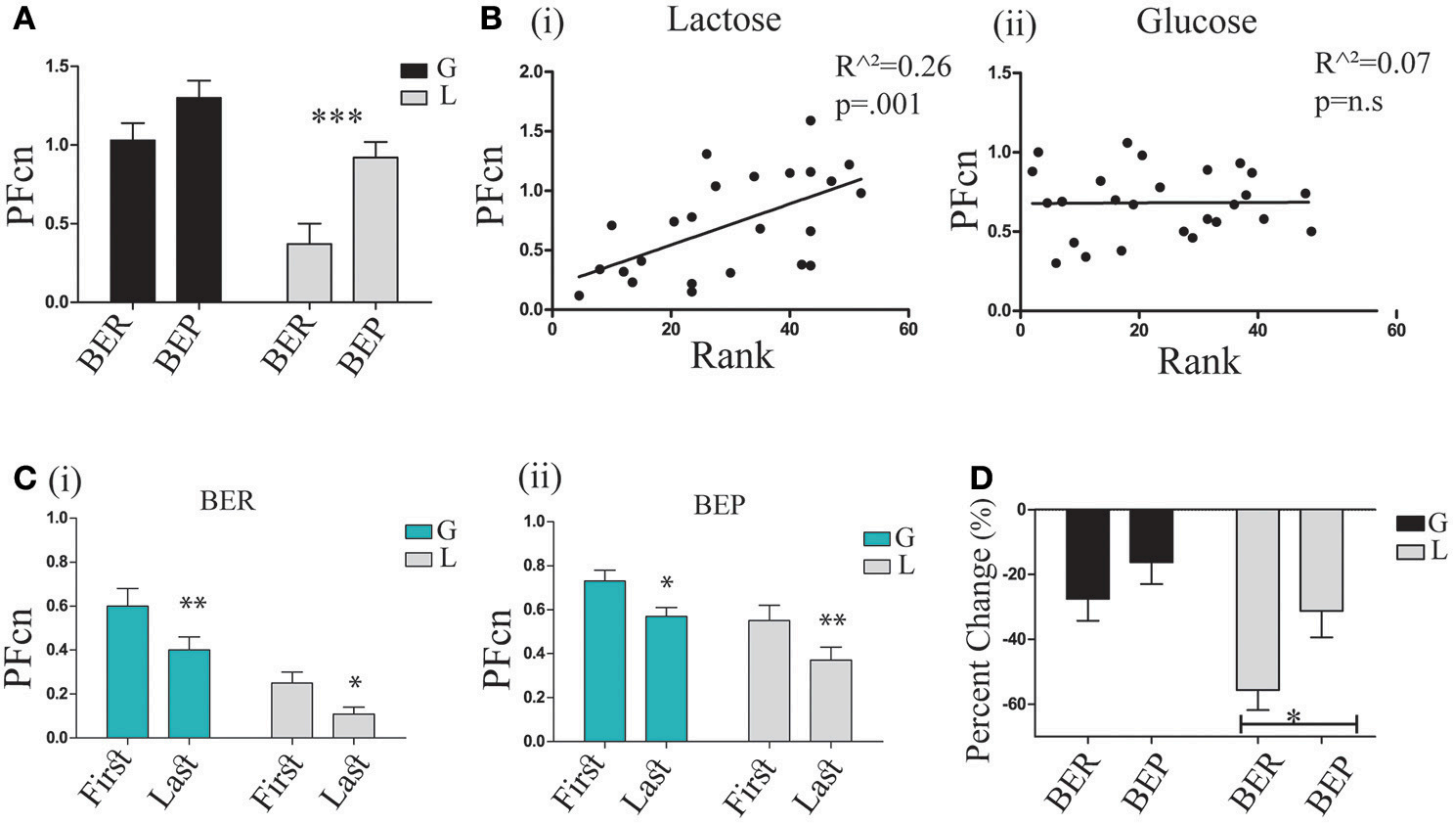
PF consumption during LCP. **(A)** Mean PFcn over total 4 h for the three conditioning tests for each group; BER-L consumed significantly less PFcn throughout LCP compared to BEP-L. **(B)** (i) Oreo cookies PFcn of lactose conditioned rats was predicted by rank order of Ensure PF intake during Phase 1 (ii) Oreo cookies PFcn of glucose conditioned rats was not predicted by rank order of Ensure PF intake during Phase 1. **(C)** ANOVA for repeated measures of the PFcn over LCP:1-3 for the first and last 2 h in both eating profile groups (i) BER and (ii) BEP. **(D)** Change in PFcn intake from the first 2 h to the last 2 h, throughout three conditioning tests (%, mean and S.E.M). ^*^p < 0.05, ^**^p < 0.005, ^***^p < 0.001.

In order to study the conditioned response (CR), PF consumption, associated with the conditioned stimulus (CS), PF (Oreo cookies), we examined the mean PFcn over the 4 h tests of the 3 LCP days in the four experimental groups (BER-L, BER-G, BEP-L and BEP-G). The results (analyzed by two-way ANOVA) are shown in Figure 4A. They revealed that the BEP (Mean = 1.09 ± 0.37) rats consumed more PF than BER (Mean = 0.76 ± 0.46) rats [*F*_(1, 29)_ = 12.58, *p* = 0.002, η^∧2^ = 0.21], and that the glucose conditioned rats (Mean = 1.16 ± 0.35) consumed more than the lactose conditioned rats [Mean = 0.72 ± 0.42, *F*_(1, 29)_ = 18.72, *p* = 0.000, η^∧2^ = 0.43]. Test of simple effects showed that BEP-L rats consumed significantly more PFcn than BER-L [*t*_(18)_ = 3.39, *p* = 0.003], without differences between BER-G and BEP-G (Figure 4A). Two regression analyses were performed separately for glucose and lactose conditioned rats with rat's rank from Phase 1 as predictor variable. The regression model for lactose-conditioned rats showed that the mean Ensure PF intake during Phase 1 predicted the mean Oreo cookies PF intake during the LCP days [*F*_(1, 24)_ = 11.38, *p* = 0.002, *R*^∧2^ = 0.34, β = 0.058], while the regression model for the glucose conditioned rats was not significant (Figure 4B). In addition, to estimate the occurrence of the abdominal discomfort, we tested the mean PFcn during the LCP days separately for the first and the last 2 h. Using ANOVA for repeated measures (Figure 4C), we found that all experimental groups decreased their intake during the last 2 h [*F*_(1, 35)_ = 21.403, *p* = 0.00, η^∧2^ = 0.42] but the lactose-conditioned groups showed the largest decrease in PF consumption [main effect for sugar: *F*_(1, 35)_ = 12.206, *p* = 0.001, η^∧2^ = 0.24, Figure 4C].

Based on these findings, we are assuming that the abdominal discomfort had occurred approximately after 2 h. Interestingly, and as hypothesized, BEPs who ingested lactose did not show a decreased PF intake in the same magnitude as BERs who ingested lactose (analyzed by *t*-tests on the differences between the PFcn in the first 2 h and the last 2 h, shown in Figure 4D).

**Figure 5:**
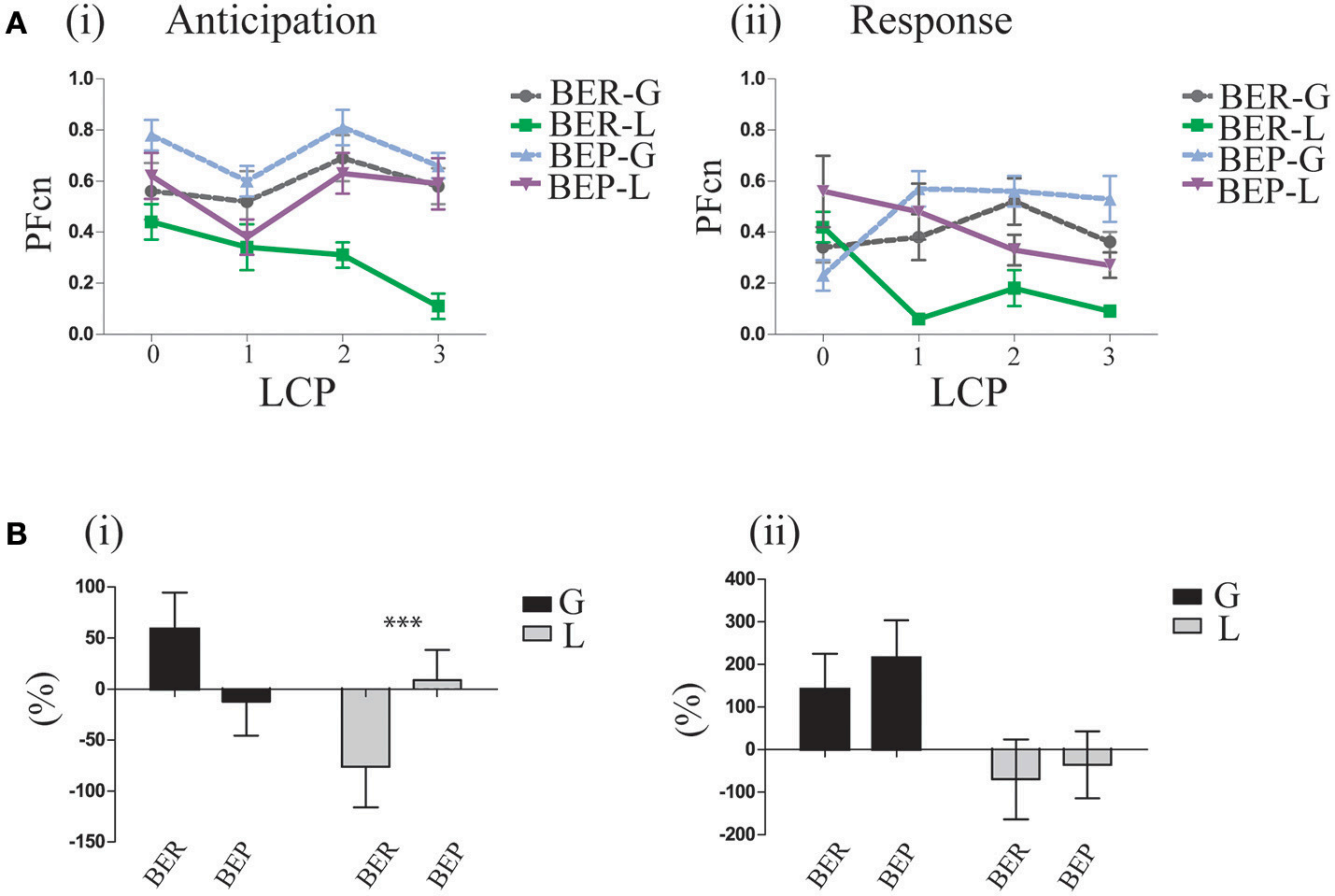
Gradual learning. **(A)** Learning process of lactose impact as reflected in the difference between LCP days in the first and last 2 h of the binge period (i) Oreo cookies PFcn of each group at the first 2 h of baseline and of the three conditioning test days (ii) PFcn of each group at the last 2 h of LCP days. **(B)** Change in PFcn from LCP-0 to LCP-3, separately for eating profiles over three conditioning tests (i) First 2 h (ii) Last 2 h (% change, mean and S.E.M). ^*^p < 0.05, ^**^p < 0.005, ^***^p < 0.001.

In order to determine if indeed the rats had learned the association between Oreo cookies PF and abdominal discomfort throughout the LCP days, we analyzed the gradual learning from baseline (LCP-0) to the rest of the LCP days for the first and last 2 h. After concluding that the abdominal discomfort is likely represented in the last 2 h, we can now refer to the first 2 h as “anticipation for aversive effect” and to the last 2 h as “response to aversive effect.” Learning curves for the anticipation period showed that after baseline, the glucose-conditioned groups and the BEP-L group did not change their PFcn, while the BER-L decreased their PFcn significantly [*F*_(3, 33)_ = 5.97, *p* = 0.002, η^∧2^ = 0.34]. These results were supported also by the percent change from LCP-0 to LCP-3 [*F*_(1, 35)_ = 5.03, *p* = 0.031, η^∧2^ = 0.21, Figure 5B] in which BER-L decreased their intake more than BEP-L [*t*_(18)_ = −2.15, *p* = 0.045] at the first 2 h while this eating profile effect was not significant for the glucose-conditioned groups.

Learning curves of the response to the aversive effect showed that both lactose-conditioned groups decreased their PF intake over time, while glucose-conditioned groups remained at high consumption [*F*_(1, 33)_ = 5.69, *p* = 0.003, η^∧2^ = 0.32] for both eating profile groups (Figure 5B).

**Figure 6:**
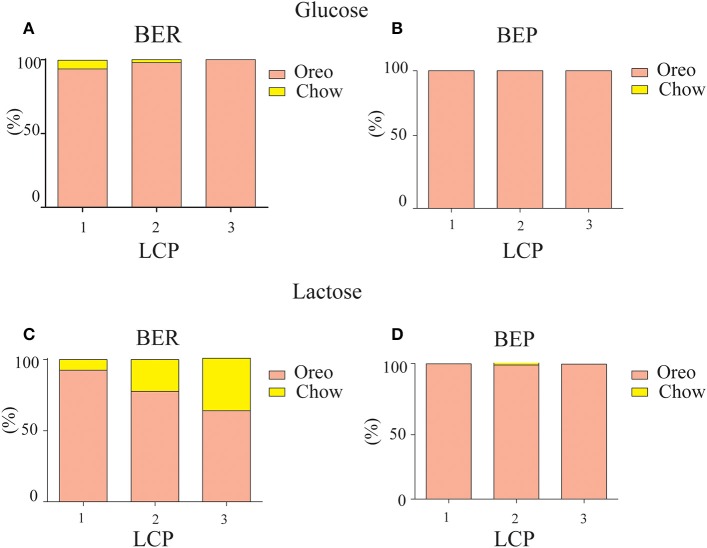
PF preference. PF preference on LCP days over the total 4 h PF binge period. **(A)** BER glucose-conditioned rats. **(B)** BEP glucose-conditioned rats. **(C)** BER lactose-conditioned rats. **(D)** BEP lactose-conditioned rats.

As shown in Figure 6, the caloric consumption of PF in all groups was higher compared to chow, indicating PF preference [*F*_(1, 35)_ = 4.47, *p* = 0.042, η^∧2^ = 0.28]. Both glucose-conditioned groups and lactose conditioned BEP rats remained with a high PF preference during LCP [*F*_(1, 35)_ = 14.84, *p* = 0.000, η^∧2^ = 0.29]. However, lactose conditioned BER rats demonstrated a relative devaluation of the PF over time [*F*_(1, 35)_ = 4.83, *p* = 0.035, η^∧2^ = 0.31; Table 1]. Thus, the decreased intake in PF during the last 2 h indeed suggests an association between the Oreo cookies and abdominal discomfort, because even though the lactose conditioned BER rats decreased their PF intake at the last 2 h, they increased their chow intake during this time, so the total caloric intake remained stable throughout the LCP days (Table 1).

**Table 1:** Solution intake and total caloric consumption.

		**Glucose mean (±S.T.D)**		**Lactose mean (±S.T.D)**	
		**BER**	**BEP**	**BER**	**BEP**
LIP-0	Time to finish solution (min)	31.6 (21.87)	38.05 (21.92)	20.14 (10.79)	29 (22.43)
	Total caloric intake (g)	34.52 (7.34)	38.7 (12.04)	34.21 (10.63)	37.44 (18.38)
LIP-1	Time to finish solution (min)	16.44 (16.7)	36.1 (23.53)	30 (21.25)	24.5 (19.42)
	Total caloric intake (g)	33.76 (19.2)	44.27 (14.16)	13.69 (11.56)	32.69 (15.73)
LIP-3	Time to finish solution (min)	11 (7.03)	17.1 (10.82)	27.85 (19.55)	25 (21.19)
	Total caloric intake (g)	38.87 (13.05)	45.16 (13.14)	11.66 (6.15)	34.01 (16.87)
Long term	Time to finish solution (min)	7.22 (3.19)	12.65 (8.68)	10.57 (9.53)	20.03 (18.19)
	Total caloric intake (g)	39.31 (19.35)	42.04 (7.51)	27 (9.31)	40.54 (10.99)

Interestingly, during the LCP days, the duration of Ensure and sugar (solution) consumption was similar for all groups (Table 1), meaning that the rats associated between the solution eating and their removal to the regular cage (with Oreo cookies and chow) and did not associate the abdominal discomfort with the solution.

In summary, rats that ingested lactose, with no regard to their eating profile, demonstrated associative learning between PF intake and abdominal discomfort during the response to aversive effect (the last 2 h). However, only BER rats reduced their PF consumption during the anticipation period (first 2 h), indicating association learning for the consequences of PF consumption.

### Phase 3: long term memory (conditioned-lactose effects)

To test for memory of the conditioning over the 3 LCP days, we performed another LCP test a week from the last LCP day. At this test, no lactose was administered, only glucose. At first encounter with Ensure and sugar solution in the empty cage, all groups of rats decreased their intake duration of the solution compared to the last day of LCP (Table 1). This decrease was significant only in the BER-L group. As shown in Table 1, no conditioned lengthening of the time until finishing the solution was detected on LCP-long term, in fact the lactose conditioned BER rats ate the solution faster than on LCP-3.

**Figure 7:**
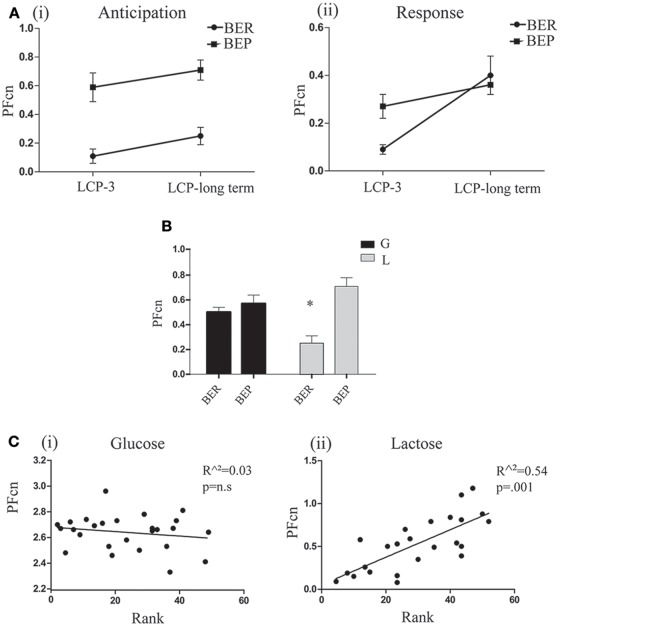
Long term test day. **(A)** (i) Mean PFcn in the first 2 h period of lactose conditioned rats during LCP-3 and LCP-long term (ii) Mean PFcn in the last 2 h period of lactose conditioned rats during LCP-3 and LCP-long term. **(B)** Mean PFcn in the first 2 h period of the long term test. **(C)** (i) Oreo cookies PFcn of glucose conditioned rats was not predicted by rank order of Ensure PF intake during Phase 1 (ii) Lactose Oreo cookies PFcn of lactose conditioned rats was predicted by rank order of Ensure PF intake during Phase 1. ^*^p < 0.05, ^**^p < 0.005, ^***^p < 0.001.

As for the PF intake in the regular cage, paired *t*-tests did not show significant differences in PFcn during the first 2 h between LCP-3 and the long term test day in both lactose conditioned groups. In addition, paired *t*-tests for the PFcn during the last 2 h between LCP-3 and the long term test day, showed significant increase of PFcn only for lactose conditioned BER rats [*t*_(6)_ = 2.48, *p* = 0.04; Figure 7A].

Two way ANOVA for PFcn on the first 2 h (anticipation for aversive effect) of the long term test day showed significant differences between eating profiles [*F*_(1, 35)_ = 13.597, *p* = 0.001, η^∧2^ = 0.22; Figure 7B]. These differences were dependent on the type of sugar (glucose/lactose) ingested at Phase 2 of this study [*F*_(1, 35)_ = 7.349, *p* = 0.01, η^∧2^ = 0.17]. For the former glucose-conditioned rats, the long term test did not reveal significant differences between eating profiles, while in the former lactose-conditioned rats, the long term test showed that BERs ate significantly less than BEPs [*t*_(18)_ = 4.2, *p* = 0.001]. Using linear regression (separately for glucose and lactose conditioned rats), we found that the PFcn in Phase 3 (long term test) can be predicted by the animal's rank ingestion of Ensure PF in Phase 1, only for rats that ingested lactose in phase 2 [*F*_(1, 23)_ = 26.54, *p* = 0.000, *r*^2^ = 0.526, β = 0.732; Figure 7C].

## Discussion

The aim of the current study was to establish an animal model of a common eating conflict in humans between intake of palatable food and its delayed consequences. The uniqueness of this model is the use of an internal pain as unconditioned stimulus (US) instead of external US such as foot shocks. The use of lower intestinal discomfort represents a step toward better understanding of daily behaviors due to its less artificial nature and its dynamic value. Another significant advantage in using internal discomfort as a US is the possibility to alter the perceived source of pain due to the time passing until the onset of the discomfort. In this way we succeeded in associating the Oreo cookies with the abdominal discomfort and not with the sugar laced solution.

The LCP model was created especially to capture the dynamic nature of decision making in complex situations such as those including both positive and negative rewards. In contrast to conditioned taste aversion or conditioned place preference protocols, this protocol allows us to portray a process of decision making regarding future behaviors and to test the differences between anticipation for an aversive event and the response to it. Other protocols that include decision making between immediate and delayed rewards focus, to our best knowledge, on different sizes of positive rewards anticipated in variable intervals. The LCP however, enables us to test decision making between an immediate reward and a future negative reward.

In this study, the division to eating profiles based on the Ensure tests (Phase 1) was reliable and consistent throughout the study. The BE-like profile in female Wistar rats was consistent even when the PF changed from a liquid (Ensure) to a solid (Oreo cookies) diet. This strengthens and adds to the previous knowledge about female Sprague-Dawley rats that transfer their profile from one PF (Oreo cookies) to a variety of solid PFs: a non-nutritive high-fat/sweet food (Oreo-like pellets), a nutritive non-fat/sweet food (Froot Loops), a non-nutritive/high-fat (Crisco) and a nutritive/high-fat food (35% high-fat pellet; Boggiano et al., 2007).

Based on our results, it can be concluded that the trait binge eaters (BEP) showed high motivation to obtain a large amount of PF even during experience of discomfort caused by lactose ingestion. The preference for PF over chow was high for all rats in this study. However, the value of the PF decreased in response to lactose ingestion only in rats that ranked low in Phase 1. We believe that these results are not due to differences in pain tolerance but to individual difference in motivation toward PF. In a study with similar craving behavior (Vanderschuren and Everitt, 2004) the differences in conditioned suppression in rats exposed to cocaine for an extended period were not due to differences in pain sensitivity or an inability to encode the CS aversive association, when using a test of conditioned freezing during the CS presentation. We also minimized the possible variability in pain sensitivity throughout the estrus cycle by using a relatively large sample size.

As expected, we found differences between eating profiles in decision making during the LCP days. BEP's behavior in this study strengthen the characterization of eating profiles described in Oswald et al. (2011). In their report, BEPs showed higher tolerance for foot shock than BERs, meaning that BEPs have high motivation toward PF, even in the face of an immediate aversive effect (Oswald et al., 2011). Our findings may further indicate that BEPs have greater DRD, in which consumption of PF, occurring despite harmful consequences, indicates pathological motivation for food (Ventura et al., 2013). Another explanation for these differences between the BE-like profiles is a possible high hedonic threshold or heightened reward sensitivity in binge eaters (Boggiano et al., 2007). Differences in sympathetic reactivity may predispose binge eaters to turn to palatable food while dealing with stress (Boggiano et al., 2007, 2015; Corwin et al., 2011; Burgess et al., 2014; Pool et al., 2015). Stress elevates corticosterone (CORT) that enhances dopamine release in the NAc that affect the motivation toward PF. high-fat diet removal caused CORT to increase (Teegarden and Bale, 2007), It may be that high fat diet removal plays as a stressor, similar to a situation of withdrawal from addictive drugs (Nava et al., 2006).

Based on differences in gradual learning between the first and the last 2 h (Figure 5), we estimated the occurrence of the abdominal discomfort at the last 2 h, between the first measure and the last measure. In this study, there was no precise index for the duration, intensity and onset time of the abdominal discomfort. We presented our interpretations regarding these issues, based on the behavioral results. Accordingly, we refer to the first 2 h as anticipation for aversive effect and to the last 2 h as the response to aversive effect. The BER-lactose conditioned rats in this study demonstrated adaptive behavior throughout the LCP days by decreasing there intake at the first 2 h, representing anticipation for the aversive effect. In contrast, BEP-L kept eating large amounts of PF similarly to the glucose-conditioned groups. Furthermore, although the BEP-L did decrease their PF intake during the last 2 h throughout the study, their decline was lower compared to BER-L. This behavioral pattern suggests that BEPs devaluate the later expected consequences of abdominal discomfort. A possible biologic explanation for these finding is the high function of the mu-opioid receptor gene in binge eaters (Kelley et al., 2003; Davis et al., 2009; Corwin et al., 2011) that increases intake of palatable food (Kelley et al., 2003) by increasing motivation to obtain a desired outcome.

We were able to successfully show the existence of long term memory for the association between PF and delayed lower abdominal discomfort. All groups of rats ate the same amount of PF during the first 2 h of the long term test day as they ate in the last LCP day, indicating anticipation for aversive effect. Interestingly, when the abdominal discomfort failed to arrive (during the last 2 h of the long term test day), only lactose conditioned BER rats increased their PFcn compared to the last LCP day. PF intake during the last 2 h for these lactose conditioned BER rats fits our hypothesis that they monitor their abdominal discomfort and consume accordingly so when the abdominal discomfort fails to emerge they increased their intake. In contrast, lactose conditioned BEP rats did not change their intake from the last LCP day (but did eat less than glucose conditioned BEP rats) even in the absence of abdominal discomfort (Figure 7A). This suggests that there are differences in the memory for the association learning in which lactose conditioned BER present an adaptive dynamic behavior that matches immediate changes in the situation, whereas lactose conditioned BEP rats do not.

A major achievement of this new LCP model is in altering the value of the conditional stimuli (CS), Oreo cookies, from safe to potentially harmful in BER rats. We assume that the PF valuation is impaired or altered in trait binge eaters. It has been recently shown (Singer et al., 2016) that there are individual variations in conditioned response (CR), even after identical pairing of conditional stimuli (CS) and unconditional stimuli (US). One suggested explanation for such variation is the motivational value given to the CS. Singer et al. (2016) suggested that it is likely that changes in the sensory quality of the CS may change the nature of the CS-US relationship (in this case; between the PF and intake implication), by dopamine signaling (intake of palatable foods elicits dopamine release from VTA cells to NAc; Roh et al., 2016). Lactose conditioned BER rats represent a flexible and adaptive cognition which involves calculation of reward value and consequences of behavior. Judging our results under these assumptions, we can say that the BER rats, and not the BEP, adopted the alternation rapidly in an adjusted manner. This lack in behavioral flexibility in BEPs, interferes with the adaption of coping in accordance to the environment (Bickel et al., 2012), implicating impulsive choice that values an immediate reward even in face of an aversive delayed (Velázquez-Sánchez et al., 2014). Therefore, we suggest that the interpretation of the “loss of control” aspect in the binge eating definition be directed to the maladaptive motivation that leads the entire decision making process in this context, and not to the behavior of overeating itself. Another possible explanation is based on Boggiano et al. (2007)'s observation that BER rats were hypersensitive to a footshock stressor. It is therefore possible that binge eaters are less affected by stress related effects of future events such as the ramifications of excessive eating and are therefore more susceptible to the influence of an immediate motivation and need for rewarding food. This type of susceptibility could potentially interfere with the process of adaptive learning and decision making.

In our opinion this study is an important step toward better understanding of individual differences in motivational behaviors, especially in the field of eating disorders and healthy body weight maintenance. This is, to our knowledge, the first study demonstrating an animal model in female Wistar rats that represents the common conflict of PF consumption and its anticipated negative, delayed effects. Furthermore, we were able to demonstrate binge eating in Wistar female rats although Sprague-Dawley female rats are at higher risk for BE and are usually in use in this kind of studies (Hildebrandt et al., 2014). The translational value of this model may be increased by assessing brain mechanisms underlying the differences reported in this paper, measures of pain receptor density in the intestine, lactase activity and gastrointestinal motility. This is not the first study that relies on pain avoidance as a signal for eating motivation (see Oswald et al., 2011) in the context of binge eating. We believe that the current model and the LCP can be used in a variety of additional ways and can include different kinds of conflicts and delayed effects, providing the opportunity to examine underlying mechanisms.

## Author contributions

LM planned and performed the experiment, analyzed the data and wrote the initial draft of the manuscript. LB contributed significantly to data analysis and manuscript writing and editing. AW supervised the study, including planning the study, assisted in data analysis and in editing the manuscript.

### Conflict of interest statement

The authors declare that the research was conducted in the absence of any commercial or financial relationships that could be construed as a potential conflict of interest.
